# Long‐Term Trends in Parkinson's Disease and Associated Mental Health Disorders: Insights From the CDC WONDER Database, 1999–2023

**DOI:** 10.1002/brb3.71190

**Published:** 2026-01-13

**Authors:** Taha Alam, Waqas Burney, Sohaima Kamal, Ahmad Kamal, Iman Osman Abufatima, Umair Ali, Muhammad Mukhlis, Aneezeh Khatri, Norina Usman, Noorulain Aqeel, Mohammed Shahabuddin Mollah, Muhammad Shaheer Bin Faheem

**Affiliations:** ^1^ Department of Medicine Dow University of Health Sciences Karachi Pakistan; ^2^ Department of Family Medicine Dignity Health Methodist Hospital Sacramento California USA; ^3^ Department of Psychiatry Napa State Hospital Napa California USA; ^4^ Department of Research California Institute of Research and Technology Davis California USA; ^5^ Department of Medicine Bannu Medical College Khyber Pakhtunkhwa Pakistan; ^6^ Department of Medicine University of Medical Sciences and Technology Khartoum Sudan; ^7^ Department of Pharmacy University of Swabi Khyber Pakhtunkhwa Pakistan; ^8^ Department of Medicine Ayub Medical College Abbottabad Pakistan; ^9^ Department of Mental Health Crisis Text line New York New York USA; ^10^ Department of Internal Medicine Adventist Hospital Lodi California USA; ^11^ Department of Psychiatry California North State University Elk Grove California USA; ^12^ Department of Psychiatry Touro University New York California USA; ^13^ Department of Psychiatry University of California San Francisco San Francisco California USA; ^14^ Department of Medicine Karachi Institute of Medical Sciences, KIMS Karachi Pakistan

**Keywords:** age‐adjusted mortality rate, CDC WONDER, mental illness, mortality trends, older adults, Parkinson's disease, United States

## Abstract

**Background:**

The U.S. population is aging with an increasing burden of Parkinson's disease (PD) and its frequent co‐occurring mental health disorders. However, mortality trends related to PD and these comorbid mental health disorders among older adults remain understudied.

**Objective:**

To examine trends in mortality due to PD and related mental health conditions among adults aged 45 and older in the United States from 1999 to 2023.

**Methods:**

We extracted mortality data for PD and mental health‐related conditions among individuals aged 45 and older from the CDC WONDER database. Age‐adjusted mortality rates (AAMRs) were calculated per 100,000 persons and stratified by sex, race/ethnicity, census region, and urbanization status. Annual percentage changes (APCs) with their 95% confidence intervals (CIs) were estimated using the Joinpoint regression program.

**Results:**

PD and its associated mental health disorders resulted in 238,378 deaths between 1999 and 2023. The AAMR increased significantly from 3.83 in 1999 to 8.49 in 2023 with an AAPC of 3.20 (*p* = 0.005). Males consistently showed higher AAMRs than females (overall AAMR male: 11.40 vs. female: 5.75). Non‐Hispanic (NH) Whites had the highest mortality rates (8.67), while NH African Americans exhibited the lowest (4.66). Crude mortality rate was the highest among older adults (21.09), reflecting the greatest burden in this population. Similarly, the mortality rates were higher in nonmetropolitan areas (8.15) than the metropolitan areas (7.72). The highest AAMR was noted in the Midwest (9.18), with a standard deviation of 14.08 in Minnesota and 5.44 in Arizona. The majority of the deaths were recorded in nursing or long‐term care facilities (52.65%).

**Conclusion:**

The increasing trend in mortality highlights the necessity of focused preventive, diagnostic, and treatment approaches for all susceptible groups.

**Abbreviations**
AAMR: age‐adjusted mortality rate
APC: annual percent change
CDC WONDER: Centers for Disease Control and Prevention Wide‐Ranging Online Data for Epidemiological Research
CI: confidence interval
CMR: crude mortality rate
ICD‐10‐CM: International Classification of Diseases, Tenth Revision, Clinical Modification
NH: non‐Hispanic
PD: Parkinson's disease

## 1 Introduction

Parkinson's disease (PD) is one of the most disabling and prevalent neurodegenerative disorders, primarily characterized by tremor, rigidity, and bradykinesia (Zafar and Yaddanapudi 2025; Kouli et al. 2018). However, a growing body of evidence emphasizes the pivotal role of nonmotor symptoms like mental illness in disease burden, reduced quality of life, and increased mortality (Lee and Koh 2015). Approximately, 40–50% of patients with PD suffer from clinically significant depression, while anxiety affects nearly one‐third of the patients during the course of the illness (Reijnders et al. 2008; Broen et al. 2016). In addition to mood disorders, psychosis, in the form of delusions and hallucinations, occurs in nearly 20–30% of the patients with advanced disease, often exacerbated by dopaminergic therapies (Reijnders et al. 2008; Ravina et al. 2007). Moreover, cognitive impairment and dementia eventually develop in nearly 80% of the patients, representing major causes of disability and mortality (Hely et al. 2008; Aarsland et al. 2017).

Over the past two decades, advances in diagnostic accuracy, population aging, and genetic and environmental factors, such as a reduction in smoking rates and an increase in exposure to pollutants and pesticides, have collectively altered the epidemiological landscape of PD and its associated conditions (Bhidayasiri et al. 2024). Concurrently, the recognition of mental disorders as integral aspects of the PD spectrum has renewed interest in researching their long‐term trends in populations (Tripathi et al. 2022; Grover et al. 2015). Understanding how these trends evolve between cohorts and through time is essential to shape prevention strategies tailored to an aging population.

Depression, anxiety, and psychosis are the most recognized psychiatric comorbidities, but their temporal course and demographic distribution in PD remain poorly clarified (Patel et al. 2023). By utilizing data from the Centers for Disease Control and Prevention's Wide‐ranging Online Data for Epidemiologic Research (CDC WONDER) database from 1999 to 2023, this article provides new data on the evolving crossroads between neurodegeneration and mental illness, framing an epidemiologic context for prevention studies and health policy.

## 2 Methods

### 2.1 Study Setting and Population

Mortality data related to PD and its associated neuropsychiatric disorders in the U.S. population aged ≥45 years were retrieved from the CDC WONDER Multiple Cause‐of‐Death (MCD) database and analyzed for the period from January 1999 to December 2023 ([Internet] 2025). Deaths were identified where PD and the specified neuropsychiatric conditions were recorded as either an underlying or contributing cause of death. We used the following ICD‐10‐CM codes: G20 for PD, and F01, F03, F22, F23, F29, F32, F33, F34, F40, and F41 for the neuropsychiatric conditions. These codes have previously been employed in multiple studies (Akhtar et al. 2025; Patel et al. 2023; Hassan et al. 2025). The ICD‑10 mental and behavioral disorder codes analyzed represent the psychiatric conditions most commonly associated with PD, namely, cognitive disorders, psychotic disorders, depressive disorders, and anxiety disorders. These codes are well‑documented in the PD literature and are the mental health conditions most frequently recorded on death certificates (Reijnders et al. 2008; Broen et al. 2016; Ravina et al. 2007; Hely et al. 2008; Aarsland et al. 2017). Other F01–F99 conditions (e.g., personality disorders, substance‑use disorders, developmental disorders) are not typically linked to PD and are rarely documented in mortality data; therefore, they were not included. Mortality rates were calculated for middle‐aged adults (45–64 years) and older adults (65–85+ years) with totals shown. This study used a publicly available dataset and did not require institutional review board approval. The reporting of this study adheres to the Strengthening the Reporting of Observational Studies in Epidemiology (STROBE) guidelines (von Elm et al. 2008).

### 2.2 Data Abstraction

Data on population sizes, place of death, demographics (sex, race/ethnicity), and regional information (urban‐rural and state) were extracted. Place of death was categorized as medical facilities, decedent's home, hospice, and nursing home/long‐term care facilities. Race/ethnicity was classified as non‐Hispanic (NH) White, NH Black or African American, NH Asian or Pacific Islander, NH American Indian or Alaska Native, and Hispanic or Latino. This information is based on data recorded on death certificates and has been used in previous analyses of the CDC WONDER database. Counties were categorized as rural (micropolitan, noncore regions) or urban (large central metro, large fringe metro, medium metro, small metro) using the 2013 National Center for Health Statistics Urban‐Rural Classification Scheme (Ingram and Franco 2013). Regions were classified into Northeast, Midwest, South, and West according to U.S. Census Bureau definitions ([Internet] 2024).

### 2.3 Statistical Analysis

Crude mortality rates (CMRs) and age‐adjusted mortality rates (AAMRs) per 100,000 population were calculated to assess national trends in deaths related to PD and its associated neuropsychiatric disorders. CMRs were calculated by dividing the number of deaths in each category by the corresponding U.S. population for that year. AAMRs were computed by adjusting deaths to the year 2000 U.S. standard population (Anderson and Rosenberg 1998). To examine long‐term mortality trends, the Joinpoint Regression Program (Joinpoint V 5.4.0.0, National Cancer Institute) was used to estimate the annual percentage change (APC) with 95% confidence intervals (CIs) (Joinpoint Regression Program [Internet] 2025). Joinpoint regression applies log‐linear models to identify significant shifts in AAMRs over time, allowing detection of periods with temporal variations. APCs were considered increasing or decreasing if the slope of change in mortality was significantly different from zero using two‐tailed *t*‐tests. Statistical significance was defined as *p* < 0.05.

## 3 Results

Between 1999 and 2023, PD and its associated mental health disorders accounted for a total of 238,378 deaths among adults aged 45 years or older in the United States (Table 1).

**TABLE 1:** Parkinson's disease and associated mental health disorders‐related deaths stratified by sex and race among adults aged ≥45 in the United States, 1999−2023.

Year	Overall	Women	Men	NH Black or African American	NH White	Hispanic or Latino	Population
**1999**	3645	1817	1828	138	3394	83	95,153,686
**2000**	6314	3097	3217	246	5822	161	96,944,389
**2001**	6666	3141	3525	263	6132	160	99,781,854
**2002**	7045	3316	3729	292	6459	165	102,217,733
**2003**	7651	3624	4027	317	7003	202	104,692,428
**2004**	7946	3700	4246	307	7258	244	107,138,553
**2005**	8272	3936	4336	342	7532	263	109,787,199
**2006**	7829	3594	4235	355	7021	277	112,380,379
**2007**	8194	3827	4367	395	7361	281	114,894,084
**2008**	8481	3861	4620	373	7626	327	117,395,131
**2009**	8428	3897	4531	382	7529	342	119,895,863
**2010**	9204	4158	5046	391	8227	397	121,757,429
**2011**	9660	4431	5229	428	8581	447	124,174,484
**2012**	9938	4412	5526	463	8808	451	126,000,296
**2013**	10,160	4449	5711	493	8927	522	127,788,037
**2014**	10,075	4396	5679	473	8835	525	129,779,643
**2015**	10,075	4412	5663	500	8755	546	131,826,832
**2016**	10,240	4378	5862	515	8890	557	133,494,018
**2017**	10,918	4706	6212	575	9452	566	135,229,289
**2018**	11,465	4896	6569	641	9872	613	136,335,528
**2019**	11,703	4796	6907	592	10,096	667	137,381,702
**2020**	15,226	6289	8937	836	12,975	987	138,429,175
**2021**	13,436	5522	7914	730	11,440	816	139,339,453
**2022**	13,402	5464	7938	684	11,459	825	140,311,934
**2023**	12,405	5055	7350	682	10,509	798	141,596,553
**Total**	238,378	105,174	133,204	11,413	209,963	11,222	3,043,725,672

### 3.1 Annual Trends

**FIGURE 1:**
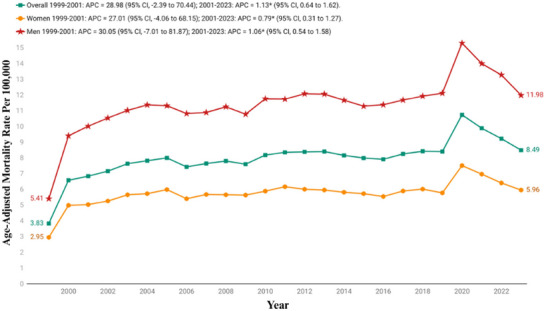
Overall and sex stratified age‐adjusted mortality rates per 100,000 for Parkinson's disease and associated mental health disorders‐related mortality in U.S. adults aged ≥45 years, 1999–2023. APC, annual percentage change; CI, confidence interval. * The annual percentage change (APC) is significantly different from zero at *α*  = 0.05.

Between 1999 and 2023, the AAMR for PD and associated mental health disorders showed an overall increasing trend. The AAMR increased significantly from 3.83 in 1999 to 8.49 in 2023 with an AAPC of 3.20 (95% CI: 0.93−5.52). From 1999 to 2001, there was a sharp rise in mortality rates, with an APC of 28.98% (95% CI: −2.39 to 70.44), indicating a steep early increase. After 2001, the trend continued to rise more gradually, with an APC of 1.13% (95% CI: 0.64−1.62), reflecting a significant but modest long‐term upward trend through 2023 (Figure 1, Tables 1 and 2, and Table S1).

**TABLE 2:** Annual percent change (APC) and average annual percent change (AAPC) of Parkinson's disease and associated mental health disorders, shown as age‐adjusted mortality rates per 100,000 among adults aged ≥45 in the United States, 1999–2023.

Year segment	APC (95% CI)	*p*‐value	AAPC (95% CI)	*p*‐value
**Overall**				
1999−2001	28.98 (−2.39 to 70.44)	0.071		
2001−2023	1.13 (0.64−1.62)	< 0.001		
1999−2023			3.20 (0.93−5.52)	0.005
**Sex**				
**Women**				
1999−2001	27.01 (−4.06 to 68.15)	0.091		
2001−2023	0.79 (0.31−1.27)	0.002		
1999−2023			2.75 (0.48−5.07)	0.017
**Men**				
1999−2001	30.05 (−7.01 to 81.87)	0.118		
2001−2023	1.06 (0.54−1.58)	< 0.001		
1999−2023			3.21 (0.49−5.99)	0.02
**Race**				
**NH‐Black or African American**				
1999−2001	34.40 (−10.09 to 100.89)	0.141		
2001−2023	1.72 (1.14−2.31)	< 0.001		
1999−2023			4.11 (0.85−7.48)	0.013
**NH‐White**				
1999−2001	27.35 (−5.04 to 70.79)	0.101		
2001−2023	1.34 (0.84−1.85)	< 0.001		
1999−2023			3.29 (0.90−5.74)	0.007
**Hispanic or Latino**				
1999−2013	4.27 (2.93−5.63)	< 0.001		
2013−2017	−5.60 (−13.75 to 3.31)	0.192		
2017−2020	14.01 (−3.61 to 34.86)	0.116		
2020−2023	−7.07 (−13.70 to 0.06)	0.052		
1999−2023			2.23 (−0.40 to 4.92)	0.097
**Age group**				
**45**−**64 years**				
1999−2023	3.42 (2.47−4.38)	< 0.001	3.42 (2.47−4.38)	< 0.001
**65**−**85+ years**				
1999−2001	33.79 (−0.44 to 79.79)	0.053		
2001−2023	0.44 (−0.05 to 0.93)	0.074		
1999−2023			2.87 (0.48−5.32)	0.018
**Urbanization**				
**Metropolitan**				
1999−2001	28.27 (−5.34 to 73.82)	0.102		
2001−2020	1.03 (0.40−1.66)	0.003		
1999−2020			3.35 (0.56−6.22)	0.018
**Nonmetropolitan**				
1999−2001	29.21 (12.19−48.82)	0.002		
2001−2018	1.40 (1.00−1.80)	< 0.001		
2018−2020	13.14 (4.13−22.94)	0.007		
1999−2020			4.86 (3.34−6.39)	< 0.001
**Census region**				
**Northeast**				
1999−2001	28.53 (−8.87 to 81.28)	0.144		
2001−2023	1.34 (0.74−1.94)	< 0.001		
1999−2023			3.36 (0.57−6.24)	0.018
**Midwest**				
1999−2001	32.55 (−2.04 to 79.35)	0.066		
2001−2023	0.86 (0.36−1.37)	0.002		
1999−2023			3.18 (0.73−5.70)	0.011
**South**				
1999−2003	10.81 (3.06−19.14)	0.009		
2003−2018	0.93 (0.12−1.75)	0.027		
2018−2021	10.86 (−3.16 to 26.90)	0.124		
2021−2023	−7.94 (−19.16 to 4.84)	0.194		
1999−2023			2.93 (0.70−5.21)	0.01
**West**				
1999−2001	39.21 (−1.58 to 96.89)	0.06		
2001−2023	−0.18 (−0.71 to 0.36)	0.495		
1999−2023			2.63 (−0.16 to 5.49)	0.065

### 3.2 Sex‐Specific Trends

Between 1999 and 2023, an overall upward trend in AAMRs was observed, with males consistently exhibiting higher rates than females throughout the study period. Among females, the AAMR increased from 2.95 (95% CI: 2.81−3.09) in 1999 to 5.96 (95% CI: 5.8−6.13) in 2023. The rate rose sharply and significantly from 1999 to 2001, with an APC of 27.01% (95% CI: −4.06 to 68.15), followed by a slower but statistically significant increase from 2001 to 2023 (APC = 0.79%; 95% CI: 0.31−1.27).

Similarly, the AAMR in males was 5.41 (95% CI: 5.16−5.66) in 1999, which increased to 11.98 (95% CI: 11.71−12.26) in 2023. The trend began with a steep rise in rates from 1999 to 2001 (APC = 30.05%; 95% CI: −7.01 to 81.87), after which it continued to increase modestly but significantly from 2001 to 2023 (APC = 1.06%; 95% CI: 0.54−1.58) (Figure 1, Tables 1 and 2, and Table S1).

### 3.3 Race‐Specific Trends

The analysis of AAMRs by race reveals significant disparities across various demographic groups. When stratified by race/ethnicity, the AAMRs were highest among NH White, followed by Hispanic or Latino, and NH African Americans (mean overall AAMR NH White: 8.67; [95% CI: 8.48−8.85]; Hispanic or Latino: 5.44; [95% CI: 4.90−5.98]; NH African Americans: 4.66; [95% CI: 4.22−5.09]).

For NH White adults, the AAMR was 4.17 (95% CI: 4.03−4.31) in 1999, and rose to 9.47 (95% CI: 9.29−9.65) in 2023. The trend showed a sharp initial rise from 1999 to 2001 with an APC of 27.35% (95% CI: −5.04 to 70.79), followed by a slower but consistent growth through 2023 (APC: 1.35%; 95% CI: 0.83−1.85).

Similarly, the AAMR for Hispanic or Latino individuals increased from 2.33 (95% CI: 1.85−2.89) in 1999 to 6.18 (95% CI: 5.75−6.62) in 2023. The trend for this population showed a complex pattern, beginning with a period of steady growth from 1999 to 2013 with an APC of 4.27% (95% CI: 2.93−5.63), followed by a notable decline from 2013 to 2017 (APC: −5.60; 95% CI: −13.75 to 3.31), and a subsequent rise between 2017 and 2020 marked by an APC of 14.01% (95% CI: −3.61 to 34.86), before finally declining in the most recent years (APC: −7.07; 95% CI: −13.70 to 0.06).

**FIGURE 2:**
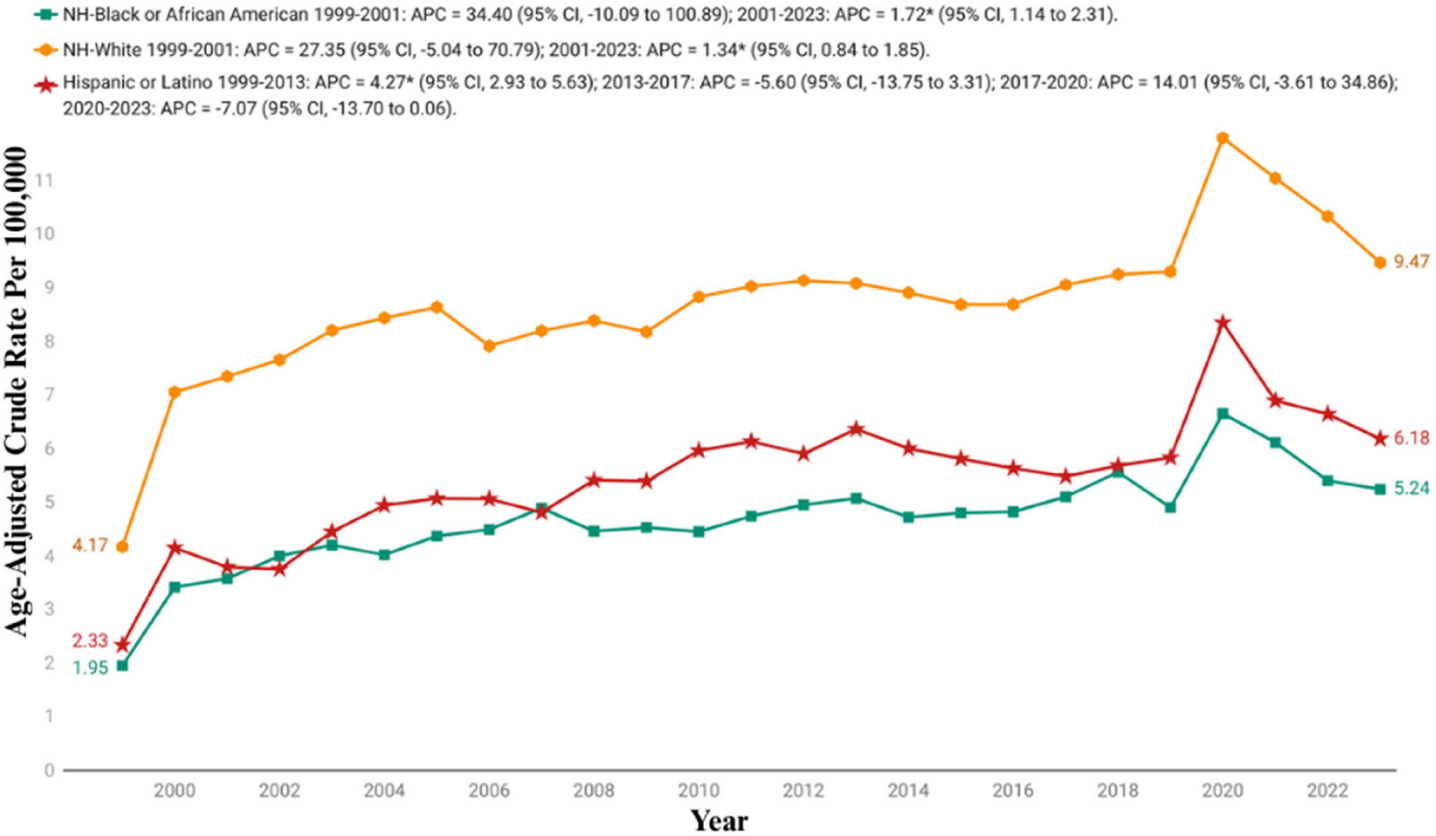
Race stratified age‐adjusted mortality rates per 100,000 for Parkinson's disease and associated mental health disorders‐related mortality in U.S. adults aged ≥45 years, 1999–2023. APC, annual percentage change; CI, confidence interval. * The annual percentage change (APC) is significantly different from zero at *α*  = 0.05.

The Black or African American group showed a steady rise in AAMRs from 1.95 (95% CI: 1.63−2.28) in 1999 to 5.24 (95% CI: 4.84−5.64) in 2023. The trend started with a significant increase from 1999 to 2001 with an APC of 34.40% (95% CI: −10.09 to 100.89), followed by a more gradual and sustained rise thereafter (APC: 1.72%; 95% CI: 1.14−2.31) (Figure 2, Tables 1 and 2, and Table S2).

### 3.4 Age Group‐Specific Trends

The age group analysis showed significant disparities. Among older adults (65−85+), the CMR increased from 10.37 (95% CI: 10.03−10.7) to 20.6 (95% CI: 20.23−20.96) between 1999 and 2023, with a mean overall CMR of 21.09 (95% CI: 20.66−21.52). The trend followed a similar pattern with rates significantly increasing from 1999 to 2001 with an APC of 33.79% (95% CI: −0.44 to 79.79), followed by a period of more gradual increase between 2001 and 2023 (APC: 0.44%; 95% CI: −0.05 to 0.93), reflecting the highest burden in this population.

**FIGURE 3:**
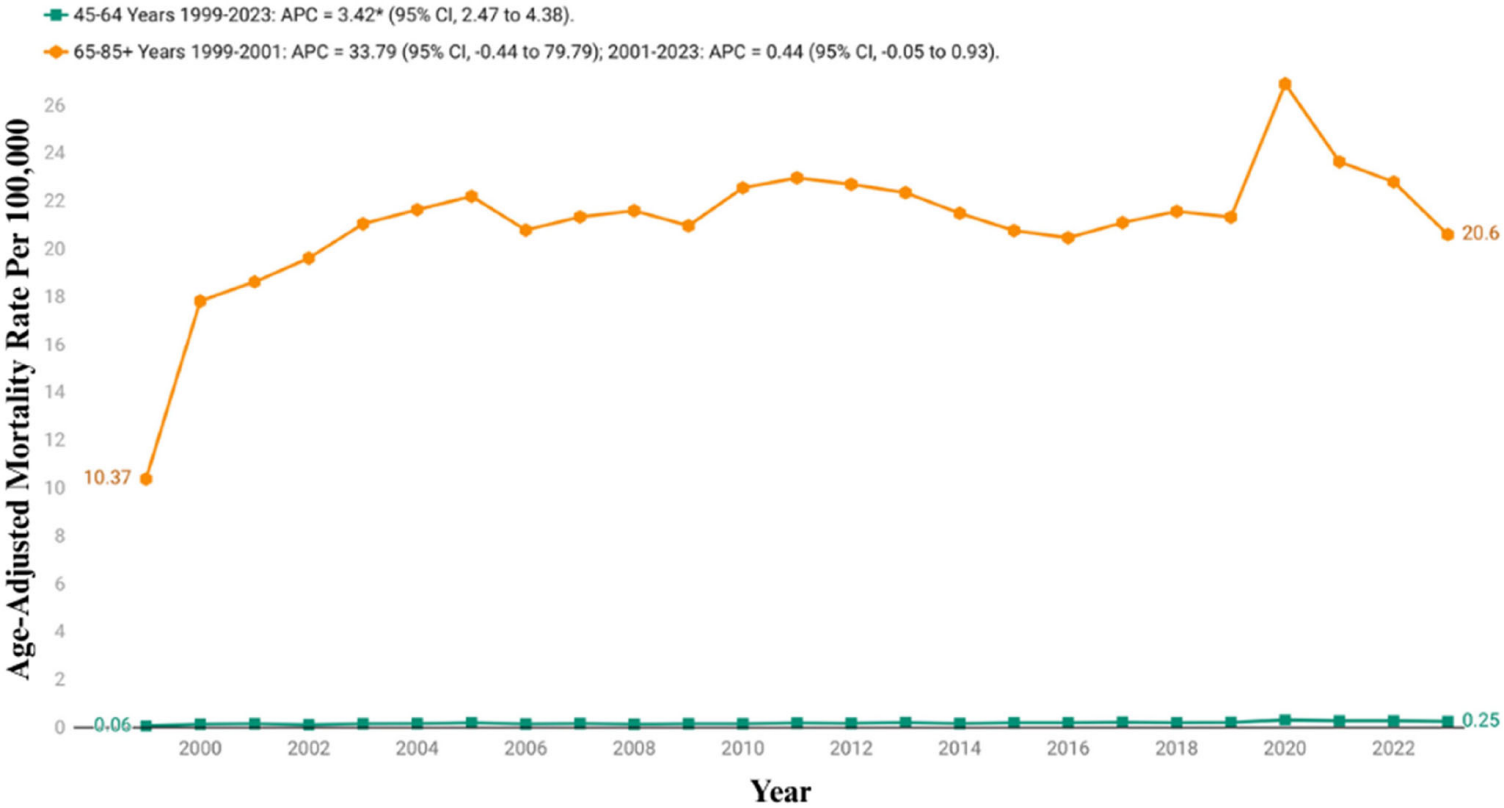
Age stratified crude mortality rates per 100,000 for Parkinson's disease and associated mental health disorders‐related mortality in U.S. adults aged ≥45 years, 1999–2023. APC, annual percentage change; CI, confidence interval. * The annual percentage change (APC) is significantly different from zero at *α*  = 0.05.

Similarly, the CMR in middle‐aged adults (45−64 years) increased from 0.06 (95% CI: 0.04−0.09) in 1999 to 0.25 (95% CI: 0.21−0.28) in 2020, with an overall CMR of 0.18 (95% CI: 0.15−0.21). The rates for this age group increased steadily throughout the study period with an AAPC of 3.42 (95% CI: 2.47−4.38) (Figure 3, Table 2, and Table S3).

### 3.5 Urbanization‐Specific Trends

By urbanization status, nonmetropolitan areas (mean overall AAMR: 8.15; 95% CI: 7.76−8.54) had higher AAMRs than metropolitan areas (mean overall AAMR: 7.72; 95% CI: 7.54−7.9). The analysis showed a consistent upward trend. For metropolitan regions, the AAMR rose from 3.82 (95% CI: 3.68−3.98) in 1999 to 10.45 (95% CI: 10.26−10.63) in 2020. The trend began with the sharpest rise from 1999 to 2001 (APC: 28.27; 95% CI: −5.34 to 73.28), which was then followed by a period of slower rise from 2001 to 2020 (APC: 1.03; 95% CI: 0.40−1.66).

**FIGURE 4:**
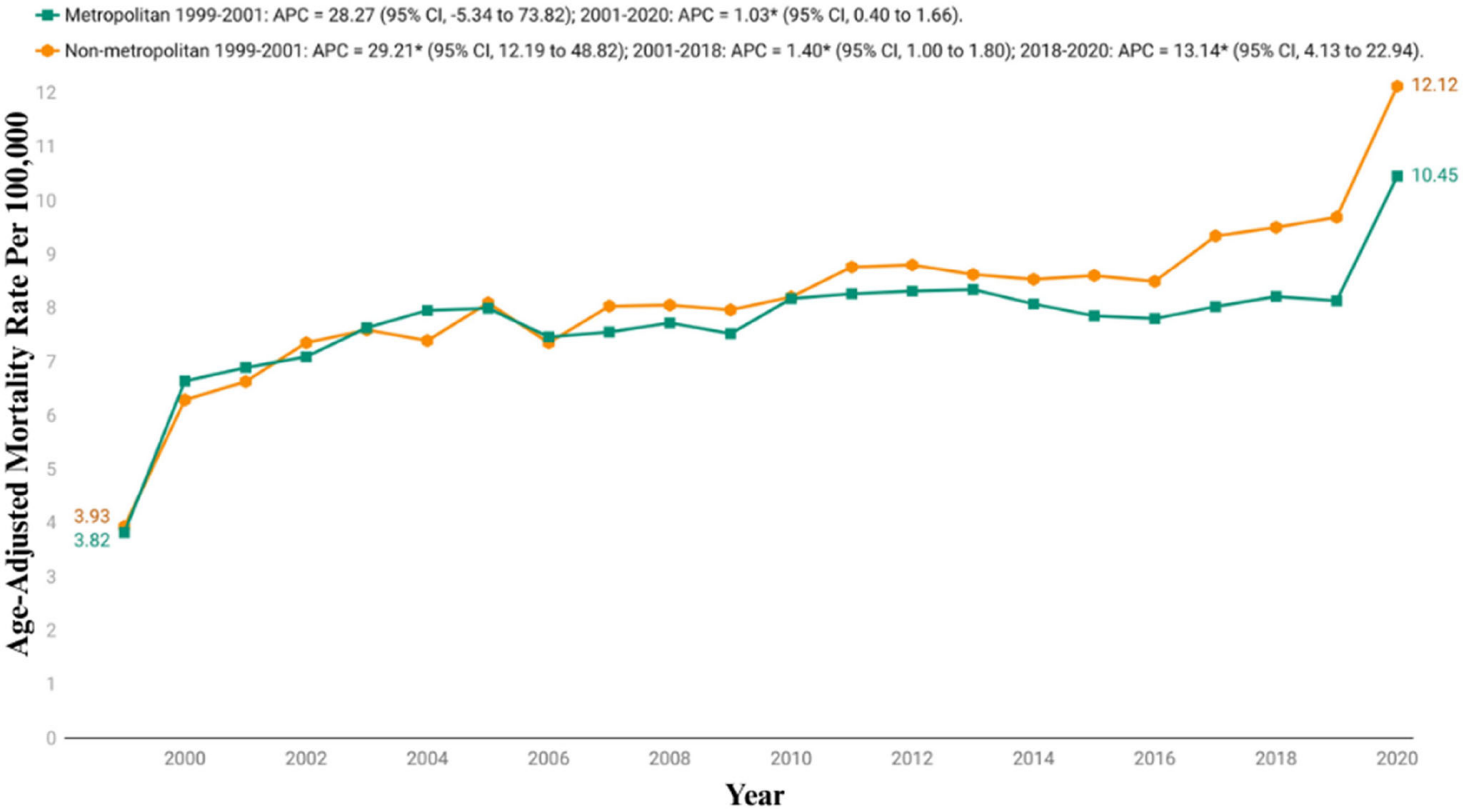
Urbanization stratified age‐adjusted mortality rates per 100,000 for Parkinson's disease and associated mental health disorders‐related mortality in U.S. adults aged ≥45 years, 1999–2020. APC, annual percentage change; CI, confidence interval. * The annual percentage change (APC) is significantly different from zero at *α*  = 0.05.

For nonmetropolitan regions, the AAMR rose from 3.93 (95% CI: 3.64−4.21) in 1999 to 12.12 (95% CI: 11.68−12.56) in 2020, with the most significant increase from 1999 to 2001 (APC: 29.21; 95% CI: 12.19−48.82), followed by a more gradual rise between 2001 and 2018 (APC: 1.40; 95% CI: 1.0−1.80), before culminating into a final surge between 2018 and 2020 (APC: 13.14; 95% CI: 4.13−22.94) (Figure 4, Table 2, and Table S4).

### 3.6 Census‐Specific Trends

Over the course of the study, the highest mortality was observed in the Midwestern region (mean AAMR: 9.18; 95% CI: 8.82−9.55), followed by the Southern (mean AAMR: 7.70; 95% CI: 7.43−7.96), Northeastern (mean AAMR: 7.58; 95% CI: 7.23−7.93), and Western regions (mean AAMR: 7.47; 95% CI: 7.12−7.81).

For the Midwest, the AAMR increased from 4.34 (95% CI: 4.07−4.61) in 1999 to 9.37 (95% CI: 9.03−9.72) in 2023. The trend showed a similar pattern with a significant increase observed between 1999 and 2001 (APC 32.56; 95% CI: −2.04 to 79.35), followed by a continued but slower rise from 2001 to 2023 (APC: 0.86%; 95% CI: 0.36−1.37).

The South had an AAMR of 4.07 (95% CI: 3.85−4.28) in 1999, which rose to 9.39 (95% CI: 9.13−9.64) in 2023. The trend revealed a rapid initial increase from 1999 to 2003 (APC: 10.81%; 95% CI: 3.06−19.14), followed by a period of slower growth from 2003 to 2018 (APC: 0.93%; 95% CI: 0.13−1.74), then a sharper rise again between 2018 and 2021 with an APC of 10.86% (95% CI: −3.16 to 26.89), before a declining from 2021 to 2023 (APC: −7.94%; 95% CI: −19.16 to 4.83).

The Northeast showed an increase in AAMR from 3.58 (95% CI: 3.32−3.84) in 1999 to 7.62 (95% CI: 7.29−7.85) in 2023, with a notable increase from 1999 to 2010 with an APC of 28.53% (95% CI: −8.87 to 81.28), followed by a gradual rise from 2001 to 2023 with an APC of 1.34% (95% CI: 0.74−1.94).

**FIGURE 5:**
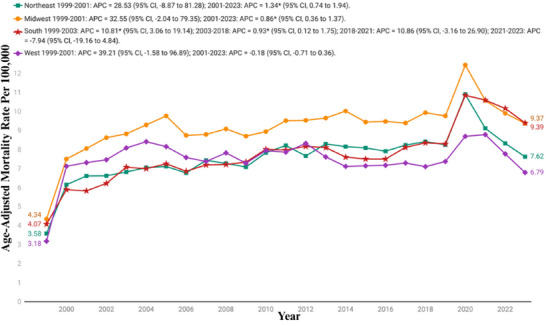
Census region stratified age‐adjusted mortality rates per 100,000 for Parkinson's disease and associated mental health disorders‐related mortality in U.S. adults aged ≥45 years, 1999–2023. APC, annual percentage change; CI, confidence interval. * The annual percentage change (APC) is significantly different from zero at *α*  = 0.05.

The AAMR for West increased from 3.18 (95% CI: 2.92−3.44) in 1999 to 6.79 (95% CI: 6.51−7.08) in 2023. The trend began with an initial rise of 39.21% (95% CI: −1.58 to 96.89) between 1999 and 2001, followed by a slight decline from 2001 to 2023 (APC: −0.18%; 95% CI: −0.71 to 0.36) (Figure 5, Table 2, and Table S5).

### 3.7 States‐Specific Trends

Death rates showed significant variations across different states, with the mean AAMRs ranging from 14.08 (95% CI: 13.46−14.69) in Minnesota to 5.44 (95% CI: 5.12−5.78) in Arizona. The states with the highest rates, which fell into the top 10th percentile, included Minnesota (mean AAMR: 14.08), Kentucky (mean AAMR: 13.80), Nebraska (mean AAMR: 13.53), Oregon (mean AAMR: 12.81), and South Carolina (mean AAMR: 11.71), while those in the lower 10th percentile included Arizona (mean AAMR: 5.44), Nevada (mean AAMR: 5.61), Louisiana (mean AAMR: 5.79), Alabama (mean AAMR: 6.06), and Hawaii (mean AAMR: 6.10) (Figure S1, Tables S6 and S7).

### 3.8 Place of Death‐Specific Trends

Information on the location of death was available for 237,944 deaths. Information on the location of death was available for 237,944 deaths. The majority of these fatalities occurred in the nursing homes or long‐term care facilities (52.65%), followed by decedents’ homes (21.05%), medical facilities (15.48%), other locations (5.70%), and hospice facilities (4.94%) (Figure S2 and Table S8).

## 4 Discussion

The long‐term mortality trajectory of PD with coexisting mental health disorders in the United States demonstrates a consistent upward pattern across the 25‐year period, aligning with global reports indicating that the dual burden of neurodegeneration and psychiatric morbidity is increasing. This rise parallels worldwide demographic aging, improved diagnostic recognition, and lengthened disease survival, factors that collectively expand the window for psychiatric comorbidities to manifest. International mortality analyses have also attributed similar increases to more complete reporting of PD on death certificates and heightened awareness of cognitive and behavioral complications. Earlier literature describing stable or declining mortality largely predated the contemporary aging surge and widespread inclusion of PD‐related dementia codes in mortality statistics (Li et al. 2025; Burchill et al. 2024). Therefore, the observed increase reflects both epidemiologic reality and improved attribution accuracy.

Increases in PD‐related deaths should not be interpreted as worsening individual disease burden. Much of the observed rise reflects population aging, growth of the PD‐prevalent population, and improved post‐diagnosis survival (Noyes et al. 2024; Peng et al. 2025). When age‐standardized, mortality trends are markedly attenuated, indicating that demographic shifts and ascertainment changes, rather than true increases in severity, substantially contribute to the pattern. Part of the apparent early rise is also influenced by coding and documentation factors. The steep rise observed between 1999 and 2001 likely reflects coding changes associated with the transition to ICD‐10 and improvements in PD documentation rather than a sudden epidemiological shift (Pan et al. 2025). Additionally, some subgroup estimates, especially among Hispanic decedents, have wide confidence intervals, and their trends should be interpreted cautiously. The overall trend corresponds with global neurologic disease forecasts, which project PD and its neuropsychiatric complications to become leading contributors to disability‐adjusted life years by 2050 (Su et al. 2025).

Even though the trend for the increase is overall in accordance with the majority of studies, the rate and period of inflection differ geographically, something that literature accounts for as due to differences in access to healthcare, exposure to environmental neurotoxins, and differences in diagnostic capacity. European and East Asian observations indicate that enhanced survival in PD patients somehow skews mortality rates by contributing to the number of people living to late phases of disease in which dementia, psychosis, and depressive syndromes accelerate the rate of functional decline. The American experience is no exception in that survival with dopaminergic treatment progresses hand in hand with increasing late‐stage death, exemplifying the two‐edged sword effect of prolonged disease control (Willis 2013; Forsaa et al. 2010).

Sex‐specific trends reveal men to consistently have greater PD‐related mortality than women, a finding consistently buttressed by population‐based registries and meta‐analyses. Biologic vulnerability through estrogenic neuroprotection, reduction of burden of oxidative stress, and dopaminergic neuroanatomic variation have all been widely hypothesized explanatory factors for the women's relative protection (Gillies et al. 2014; Bourque et al. 2009). Outside of biological factors, however, gendered health‐seeking behavior and comorbid patterns also play a significant role. Men are most likely to delay healthcare use, have greater cardiovascular and metabolic comorbidities, and have lower adherence to antidepressant use, all of which increase mortality risk (Fullard et al. 2018; Maccarrone et al. 2024). Simultaneously, women experience a higher prevalence of affective symptoms and motor disorder treatment, yet are rewarded with greater continuity of psychiatric care. Descriptive accounts attribute the narrowing male–female mortality differentials over the past decade to improved earlier diagnosis among men, gender‐neutral provision of neurologic services, and greater access to community‐based management programs. However, with fixed sex differences, the persistent racial differences reassert the convergence of sociocultural determinants of health and biological susceptibility to a single etiology (Dahodwala et al. 2009; Aamodt et al. 2023).

Racial disparities remain a characteristic of PD mortality. In line with previous studies, NH White adults have consistently shown the highest reported deaths, but rising trends in NH Black and Hispanic populations in later years indicate shifting epidemiologic trends (Hadidchi et al. 2025; Sokhal et al. 2024). The initially higher NH White death rate was previously attributed to greater completion of diagnosis capture and longer survival, permitting PD inclusion as a cause of death. But recent data indicate that structural health disparities in medical treatment, unequal access to neurology specialists, and underdiagnosis in minority groups have masked the full extent among non‐White populations (di Luca et al. 2023; Harris et al. 2023). Hospitalist studies in minorities establish that NH Black individuals are referred at later stages, are gaining fewer dopaminergic prescriptions, and have lower access to deep‐brain stimulation or cognitive therapy (Hemming et al. 2011). The comparatively lower PD‐related mortality observed among NH Black and Hispanic groups should not be viewed as evidence of reduced disease burden, but rather interpreted within the context of longstanding disparities in access to neurologic care, delayed diagnostic pathways, and differential documentation practices that continue to shape how PD and its neuropsychiatric manifestations are ultimately captured in mortality records (Aamodt et al. 2023). Hispanic groups, despite having a lower absolute prevalence, show an upward trend with increased life expectancy, more metabolic risk factors, and increased awareness of neuropsychiatric symptoms within bilingual healthcare systems. These results are in harmony with global evidence unmasking disparities in diagnosis, continuity of treatment, and care support as determinants of more powerful patterns of mortality than biological variation. As a group, the racial slopes of our model register an interaction of augmented ascertainment with persistent systemic disparity (Xie et al. 2021).

Age stratification notes that the highest incidence of PD comorbid for mental disorders is observed among the elderly, with the highest prevalence being those over 75 years. The trend follows global literature affirming older age as the optimal predictor of death from PD. Earlier falls in advanced ages have been turned around in recent years since they are a reflection of population aging, improved longevity, and extended survival with more comorbidities. Elderly patients are also vulnerable to accelerated neurodegenerative course, compromised drug responsiveness, and greater vulnerability to cognitive impairment, psychosis, and depression, all factors that further increase mortality (Ryu et al. 2023; Gonzalez et al. 2022). European and Japanese research also describes that, although therapeutic improvement in the past decreased PD mortality, long‐term survival paradoxically causes long‐term exposure to neuropsychiatric side effects, frailty, and institutional dependency. Younger age groups, with lower mortality, have exhibited progressively higher increases, which were ascribed to the earlier onset of PD, environmental exposure to neurotoxins, and metabolic factors related to lifestyle. The increasing trend in all strata of age is in agreement with the hypothesis that the rise in mortality is systemic (Su et al. 2025; Lampropoulos et al. 2022).

Urban gradients reflect variation in healthcare facilities and environmental exposure. Greater nonmetropolitan mortality is in agreement with earlier U.S. research indicating rural residents to have deterrents to neurologic and psychiatric care, fewer movement‐disorder specialists, and increased diagnostic delay. Limited telemedicine adoption and transportation constraints also worsen the delay in treatment initiation (Pereira et al. 2024; Maclagan et al. 2023). Rural settings also impose other risks through prolonged exposure to pesticides, commonly cited as a PD risk factor within these agricultural settings. Urban settings, however, though resource affluent, reflect increasing mortality with aging urban populations, socioeconomic strain, and under‐capitalized long‐term care facilities. This rural‐urban doubling back of death curves in subsequent years is also reflected in reports in literature about health‐system overload and poor integration of neurology with mental healthcare, especially where there is population density (Shekhar et al. 2024; Hancock et al. 2008). Regional diversity also puts national diversity into perspective. The Midwest and South have the highest PD mortality year after year as would be anticipated with evidence for these areas having lower density of neurologic experts, greater chronic disease burden, and greater environmental risk exposure. West, however, has relatively uniform mortality until recent acceleration, as would be anticipated in the demographic growth, in‐migration of older individuals, and increased diagnostic intensity. Northeast fluctuation is evocative of compact healthcare centers but an old population with persistence of disease. These observations agree with earlier analysis showing variability in the territorial distribution of resources, medical manpower distribution, and socioeconomic setting to dominate over intrinsic disease biology. Of particular mention are those with highly developed academic neurology departments and highly developed mental health networks with a higher percentage of mortality documented, mirroring the effect of system readiness at this level (Hadidchi et al. 2025; Su et al. 2025).

Place patterns also serve to indicate that most of the PD‐related psychiatric comorbidity mortality presents in nursing home and long‐term care. This pattern aligns with evidence showing that older individuals with advanced PD and accompanying cognitive or affective impairment commonly transition to institutional care late in the disease course (Garon et al. 2025). This distribution reflects where individuals with advanced PD commonly receive end‐of‐life care and does not imply differences in care quality, which cannot be assessed from mortality data alone. Hospital death, proportionate as it is, is associated with episodes of acute decompensation instead of terminal decline. Home death, proportionate as it is, is a harbinger of impending disparity in hospice or caregiver availability. Both findings indicate the range of community‐based independence to one of institutional dependence that defines the late‐stage progression of PD with mental health comorbidity (Phillips et al. 2023; Wilson et al. 2024).

In summary, all these stratified trends point to one message: the ongoing increase in PD mortality from psychiatric illness is met with an aging population, skewed neurology service distribution, and exclusion of mental health treatment from chronic illness management. Spatial and demographic variation indicate structural rather than biological determinants, which are predictive of equal access to integrated neurological and psychiatric care being the pre‐eminent predictor of survival in this mounting legion of patients.

## 5 Limitations

The analysis is limited by a number of methodological concerns. First, the death certificate‐based mortality reports likely underestimate the true burden of PD and associated psychiatric disorders because of misclassification, state‐to‐state variation in cause‐of‐death reporting, and inconsistent documentation of mental health conditions, particularly when they are not the immediate or underlying cause of death. Second, there is no clinical validation for psychiatric ICD‐10 codes, so changes in documentation may partly account for rising trends and further contribute to the underestimation of true psychiatric comorbidity. Therefore, these trends should be interpreted as hypothesis‐generating. Third, the ecological design of CDC WONDER data precludes individual‐level adjustment for variables such as socioeconomic status, disease duration, medication compliance, and access to neurologic or psychiatric care. Fourth, in this study, some data in the race category (NH American Indians or Alaska Natives and NH Asians or Pacific Islanders) were missing or suppressed for unexplained reasons, and the availability of urbanization statistics only through 2020 further limited the complete evaluation of recent post‐pandemic urban‐rural mortality patterns. Despite these limitations, national vital statistics data remain a standard tool for population‐level mortality surveillance, and the temporal patterns observed here still provide meaningful insight.

## 6 Conclusion

PD mortality with psychiatric comorbidity is a convergence point of aging, systemic disparities, and fragmentation of healthcare. The flip‐flop of inroads into mortality rates over four decades is a testament to growing healthcare burdens imposed by neurodegenerative disease on overloaded healthcare systems. Continuing disparities by geography, place, and age only contribute to the fact that survival is increasingly tied to the alignment of mental health and neurologic resources. Central to confronting this growing public health threat is increasing access to multimodal, equitable care.

## Author Contributions

**Taha Alam**: conceptualization, data curation, formal analysis, supervision, investigation, methodology, project administration, writing – original draft, writing – review and editing, visualization. **Waqas Burney**: formal analysis, data curation, project administration, investigation, writing – original draft, writing – review and editing. **Sohaima Kamal**: formal analysis, data curation, investigation, writing – original draft, writing – review and editing. **Ahmad Kamal**: investigation, writing – original draft. **Iman Osman Abufatima**: investigation, methodology, writing – original draft, writing – review and editing. **Umair Ali**: investigation, methodology, writing – original draft. **Muhammad Mukhlis**: investigation, methodology, writing – original draft. **Aneezeh Khatri**: supervision, investigation, project administration, writing – review and editing. **Norina Usman**: supervision, investigation, project administration, writing – original draft, writing – review and editing. **Noorulain Aqeel**: validation, project administration, writing – original draft, writing – review and editing. **Mohammed Shahabuddin Mollah**: supervision, validation, project administration, writing – review and editing. **Muhammad Shaheer Bin Faheem**: validation, writing – review and editing.

## Funding

The authors have nothing to report.

## Conflicts of Interest

All authors have no relevant financial or nonfinancial interests to disclose.

## Study Participants

The research is based on deidentified human death certificate data from the CDC WONDER database. https://wonder.cdc.gov/mcd.html.

## Consent and Ethical Approval

The research is based on deidentified data and does not require the consent of the participants or ethical approval.

## Supporting information



Supplementary Material

## Data Availability

The datasets generated and/or analyzed during the current study are available from the corresponding author on reasonable request.
