# Supplementary material for: Long‐Term Trends in Parkinson's Disease and Associated Mental Health Disorders: Insights From the CDC WONDER Database, 1999–2023
Source: Brain Behav. 2026 Jan 13;16(1):e71190. doi: 10.1002/brb3.71190 (PMC12796856; doi:10.1002/brb3.71190)
Supplement: Supplementary file 1 — Supplementary Material [file BRB3-16-e71190-s001.docx]

**SUPPLEMENTARY MATERIAL**

**Long-Term Trends in Parkinson’s Disease and Associated Mental Health Disorders: Insights from the CDC WONDER Database, 1999–2023**

Taha Alam, MBBS; Waqas Burney, MD; Sohaima Kamal, MBBS; Ahmad Kamal, MBBS; Iman Osman Abufatima, MBBS; Umair Ali, MBBS; Muhammad Mukhlis, MBBS; Aneezeh Khatri, BA; Norina Usman, MD; Noorulain Aqeel, MD; Mohammed Shahabuddin Mollah, MD; Muhammad Shaheer Bin Faheem, MBBS

**Supplemental Table 1:** Overall and sex-stratified trends in Parkinson’s disease and associated mental health disorders related mortality, shown as age-adjusted mortality rates per 100,000 among adults aged ≥45 in the United States, 1999–2023.

**Supplemental Table 2:** Race-stratified trends in Parkinson’s disease and associated mental health disorders related mortality, shown as age-adjusted mortality rates per 100,000 among adults aged ≥45 in the United States, 1999–2023.

**Supplemental Table 3:** Age group–stratified trends in Parkinson’s disease and associated mental health disorders related mortality, shown as crude mortality rates per 100,000 among adults aged ≥45 in the United States, 1999–2023.

**Supplemental Table 4:** Urbanization-stratified trends in Parkinson’s disease and associated mental health disorders related mortality, shown as age-adjusted mortality rates per 100,000 among adults aged ≥45 in the United States, 1999–2020.

**Supplemental Table 5:** Census region–stratified trends in Parkinson’s disease and associated mental health disorders mortality, shown as age-adjusted mortality rates per 100,000 among adults aged ≥45 in the United States, 1999–2023.

**Supplemental Table 6:** State- trends in Parkinson’s disease and associated mental health disorders mortality, shown as age-adjusted mortality rates per 100,000 among adults aged ≥45 in the United States, 1999–2023.

**Supplemental Table 7:** State- trends in Parkinson’s disease and associated mental health disorders related mortality, shown as absolute number of deaths among adults aged ≥45 in the United States, 1999–2023.

**Supplemental Table 8:** Percentage distribution of place of death in the United States (1999–2023), with Parkinson’s disease and associated mental health disorders related mortality highlighted.

**Supplementary Figure 1:** State stratified age-adjusted mortality rates per 100,000 for Parkinson’s disease and associated mental health disorders related mortality in United States adults aged ≥45 years, 1999-2023.

**Supplementary Figure 2:** Percentage distribution of place of death in the United States (1999–2023), with Parkinson’s disease and associated mental health disorders related mortality highlighted.

**Supplemental Table 1:** Overall and sex-stratified trends in Parkinson’s disease and associated mental health disorders related mortality, shown as age-adjusted mortality rates per 100,000 among adults aged ≥45 in the United States, 1999–2023.

| **Year** | **Age-Adjusted Mortality Rate (95% CI)** | | | |
| --- | --- | --- | --- | --- |
|  | **Overall** | **Women** | **Men** |  |
| **1999** | 3.83 (3.71–3.96) | 2.95 (2.81–3.09) | 5.41 (5.16– 5.66) |  |
| **2000** | 6.58 (6.42–6.74) | 4.98 (4.80–5.15) | 9.40 (9.07– 9.73) |  |
| **2001** | 6.84 (6.68–7.01) | 5.03 (4.85–5.21) | 10.01 (9.67–10.34) |  |
| **2002** | 7.16 (6.99–7.32) | 5.26 (5.08–5.44) | 10.53 (10.19–10.87) |  |
| **2003** | 7.63 (7.46–7.80) | 5.66 (5.48–5.85) | 11.01 (10.67–11.36) |  |
| **2004** | 7.82 (7.65–8.00) | 5.73 (5.55–5.92) | 11.36 (11.01–11.70) |  |
| **2005** | 8.00 (7.83–8.18) | 5.99 (5.80–6.18) | 11.31 (10.97–11.65) |  |
| **2006** | 7.43 (7.27–7.60) | 5.41 (5.23–5.58) | 10.81 (10.48–11.13) |  |
| **2007** | 7.64 (7.47–7.80) | 5.68 (5.50–5.87) | 10.88 (10.56–11.20) |  |
| **2008** | 7.80 (7.63–7.96) | 5.66 (5.48–5.84) | 11.24 (10.91–11.56) |  |
| **2009** | 7.60 (7.44–7.76) | 5.64 (5.46–5.82) | 10.77 (10.46–11.09) |  |
| **2010** | 8.18 (8.01–8.35) | 5.89 (5.71–6.07) | 11.75 (11.43–12.08) |  |
| **2011** | 8.35 (8.18–8.51) | 6.17 (5.99–6.36) | 11.73 (11.41–12.05) |  |
| **2012** | 8.38 (8.21–8.55) | 6.01 (5.83–6.19) | 12.07 (11.75–12.39) |  |
| **2013** | 8.40 (8.24–8.56) | 5.96 (5.79–6.14) | 12.05 (11.74–12.37) |  |
| **2014** | 8.16 (8.00–8.32) | 5.82 (5.64–5.99) | 11.66 (11.36–11.97) |  |
| **2015** | 7.99 (7.83–8.14) | 5.73 (5.56–5.91) | 11.28 (10.98–11.57) |  |
| **2016** | 7.91 (7.76–8.07) | 5.55 (5.38–5.72) | 11.37 (11.08–11.66) |  |
| **2017** | 8.25 (8.09–8.40) | 5.90 (5.73–6.08) | 11.67 (11.37–11.96) |  |
| **2018** | 8.42 (8.27–8.58) | 6.02 (5.85–6.19) | 11.92 (11.63–12.21) |  |
| **2019** | 8.40 (8.25–8.55) | 5.78 (5.62–5.95) | 12.11 (11.83–12.40) |  |
| **2020** | 10.73 (10.56–10.90) | 7.51 (7.32–7.69) | 15.29 (14.97–15.61) |  |
| **2021** | 9.88 (9.71–10.04) | 6.97 (6.79–7.16) | 13.99 (13.68–14.30) |  |
| **2022** | 9.22 (9.07–9.38) | 6.41 (6.23–6.58) | 13.27 (12.97–13.56) |  |
| **2023** | 8.49 (8.34–8.64) | 5.96 (5.80–6.13) | 11.98 (11.71–12.26) |  |

**Supplemental Table 2:** Race-stratified trends in Parkinson’s disease and associated mental health disorders related mortality, shown as age-adjusted mortality rates per 100,000 among adults aged ≥45 in the United States, 1999–2023.

| **Year** | **Age-Adjusted Mortality Rate (95% CI)** | | |
| --- | --- | --- | --- |
|  | **NH Black or African American** | **NH White** | **Hispanic or Latino** |
| **1999** | 1.95 (1.63–2.28) | 4.17 (4.03–4.31) | 2.33 (1.85– 2.89) |
| **2000** | 3.41 (2.98–3.84) | 7.05 (6.87–7.23) | 4.15 (3.50– 4.81) |
| **2001** | 3.57 (3.13–4.00) | 7.34 (7.16–7.53) | 3.79 (3.19– 4.39) |
| **2002** | 4.00 (3.54–4.46) | 7.65 (7.46–7.84) | 3.75 (3.17– 4.33) |
| **2003** | 4.20 (3.73–4.66) | 8.20 (8.00–8.39) | 4.45 (3.83– 5.07) |
| **2004** | 4.02 (3.57–4.48) | 8.43 (8.24–8.63) | 4.94 (4.31– 5.57) |
| **2005** | 4.37 (3.91–4.84) | 8.63 (8.44–8.83) | 5.07 (4.44– 5.69) |
| **2006** | 4.49 (4.02–4.95) | 7.91 (7.73–8.10) | 5.06 (4.46– 5.67) |
| **2007** | 4.89 (4.40–5.37) | 8.19 (8.01–8.38) | 4.81 (4.24– 5.38) |
| **2008** | 4.46 (4.01–4.92) | 8.38 (8.19–8.57) | 5.41 (4.82– 6.00) |
| **2009** | 4.53 (4.07–4.98) | 8.17 (7.99–8.36) | 5.39 (4.81– 5.96) |
| **2010** | 4.45 (4.01–4.90) | 8.82 (8.63–9.01) | 5.96 (5.37– 6.55) |
| **2011** | 4.74 (4.29–5.19) | 9.02 (8.83–9.21) | 6.13 (5.56– 6.71) |
| **2012** | 4.95 (4.50–5.41) | 9.13 (8.94–9.32) | 5.90 (5.35– 6.45) |
| **2013** | 5.07 (4.62–5.52) | 9.08 (8.89–9.27) | 6.36 (5.81– 6.91) |
| **2014** | 4.72 (4.29–5.14) | 8.90 (8.71–9.08) | 6.00 (5.48– 6.52) |
| **2015** | 4.80 (4.38–5.23) | 8.68 (8.49–8.86) | 5.81 (5.32– 6.30) |
| **2016** | 4.82 (4.39–5.24) | 8.68 (8.49–8.86) | 5.63 (5.16– 6.10) |
| **2017** | 5.10 (4.67–5.52) | 9.05 (8.86–9.23) | 5.48 (5.03– 5.94) |
| **2018** | 5.56 (5.12–6.00) | 9.25 (9.07–9.44) | 5.68 (5.23– 6.13) |
| **2019** | 4.90 (4.50–5.30) | 9.30 (9.12–9.48) | 5.83 (5.38– 6.28) |
| **2020** | 6.65 (6.19–7.11) | 11.79 (11.58–11.99) | 8.34 (7.82– 8.87) |
| **2021** | 6.11 (5.66–6.56) | 11.04 (10.84–11.25) | 6.89 (6.41– 7.37) |
| **2022** | 5.40 (4.99–5.82) | 10.33 (10.14–10.52) | 6.64 (6.18– 7.09) |
| **2023** | 5.24 (4.84–5.64) | 9.47 (9.29–9.65) | 6.18 (5.75– 6.62) |

**Supplemental Table 3:** Age group–stratified trends in Parkinson’s disease and associated mental health disorders related mortality, shown as crude mortality rates per 100,000 among adults aged ≥45 in the United States, 1999–2023.

| **Year** | **Crude Mortality Rate (95% CI)** | |
| --- | --- | --- |
|  | **45-64 years** | **65-85+ years** |
| **1999** | 0.06 (0.04–0.09) | 10.37 (10.03–10.70) |
| **2000** | 0.13 (0.11–0.17) | 17.81 (17.36–18.25) |
| **2001** | 0.15 (0.12–0.18) | 18.62 (18.17–19.07) |
| **2002** | 0.11 (0.09–0.14) | 19.62 (19.16–20.08) |
| **2003** | 0.15 (0.12–0.17) | 21.05 (20.58–21.53) |
| **2004** | 0.16 (0.13–0.19) | 21.64 (21.16–22.12) |
| **2005** | 0.19 (0.15–0.22) | 22.20 (21.72–22.68) |
| **2006** | 0.14 (0.11–0.16) | 20.79 (20.32–21.25) |
| **2007** | 0.16 (0.13–0.19) | 21.34 (20.87–21.81) |
| **2008** | 0.13 (0.11–0.16) | 21.60 (21.14–22.06) |
| **2009** | 0.15 (0.12–0.17) | 20.97 (20.52–21.42) |
| **2010** | 0.15 (0.12–0.18) | 22.55 (22.09–23.02) |
| **2011** | 0.18 (0.15–0.21) | 22.97 (22.51–23.44) |
| **2012** | 0.17 (0.15–0.20) | 22.70 (22.25–23.15) |
| **2013** | 0.20 (0.17–0.23) | 22.35 (21.91–22.79) |
| **2014** | 0.16 (0.14–0.19) | 21.49 (21.07–21.91) |
| **2015** | 0.19 (0.16–0.22) | 20.77 (20.36–21.17) |
| **2016** | 0.19 (0.16–0.22) | 20.47 (20.07–20.87) |
| **2017** | 0.22 (0.19–0.25) | 21.10 (20.70–21.50) |
| **2018** | 0.19 (0.16–0.22) | 21.57 (21.17–21.96) |
| **2019** | 0.21 (0.18–0.24) | 21.33 (20.94–21.71) |
| **2020** | 0.31 (0.27–0.35) | 26.89 (26.46–27.32) |
| **2021** | 0.28 (0.25–0.32) | 23.64 (23.23–24.04) |
| **2022** | 0.28 (0.24–0.31) | 22.80 (22.41–23.19) |
| **2023** | 0.25 (0.21–0.28) | 20.60 (20.23–20.96) |

**Supplemental Table 4:** Urbanization-stratified trends in Parkinson’s disease and associated mental health disorders related mortality, shown as age-adjusted mortality rates per 100,000 among adults aged ≥45 in the United States, 1999–2020.

| **Year** | **Age-Adjusted Rate (95% CI)** | |
| --- | --- | --- |
|  | **Metropolitan** | **Non-metropolitan** |
| **1999** | 3.82 (3.68–3.96) | 3.93 (3.64–4.21) |
| **2000** | 6.64 (6.46–6.82) | 6.29 (5.93–6.65) |
| **2001** | 6.89 (6.71–7.08) | 6.63 (6.26–7.00) |
| **2002** | 7.09 (6.90–7.27) | 7.35 (6.97–7.74) |
| **2003** | 7.63 (7.44–7.82) | 7.59 (7.20–7.98) |
| **2004** | 7.95 (7.76–8.14) | 7.39 (7.01–7.78) |
| **2005** | 7.99 (7.80–8.18) | 8.09 (7.68–8.49) |
| **2006** | 7.46 (7.28–7.64) | 7.35 (6.97–7.73) |
| **2007** | 7.55 (7.37–7.74) | 8.03 (7.64–8.43) |
| **2008** | 7.72 (7.53–7.90) | 8.05 (7.66–8.45) |
| **2009** | 7.52 (7.34–7.70) | 7.96 (7.57–8.36) |
| **2010** | 8.17 (7.99–8.36) | 8.20 (7.80–8.59) |
| **2011** | 8.26 (8.08–8.45) | 8.76 (8.36–9.17) |
| **2012** | 8.31 (8.13–8.50) | 8.80 (8.40–9.21) |
| **2013** | 8.34 (8.16–8.52) | 8.62 (8.22–9.02) |
| **2014** | 8.07 (7.89–8.24) | 8.53 (8.14–8.93) |
| **2015** | 7.85 (7.68–8.02) | 8.60 (8.21–8.99) |
| **2016** | 7.80 (7.63–7.97) | 8.49 (8.11–8.88) |
| **2017** | 8.02 (7.85–8.19) | 9.34 (8.94–9.74) |
| **2018** | 8.21 (8.04–8.38) | 9.50 (9.10–9.90) |
| **2019** | 8.13 (7.96–8.29) | 9.69 (9.29–10.09) |
| **2020** | 10.45 (10.26–10.63) | 12.12 (11.68–12.56) |

**Supplemental Table 5:** Census region–stratified trends in Parkinson’s disease and associated mental health disorders mortality, shown as age-adjusted mortality rates per 100,000 among adults aged ≥45 in the United States, 1999–2023.

| **Year** | **Age Adjusted Mortality Rate (95% CI)** | | | |
| --- | --- | --- | --- | --- |
|  | **Northeast** | **Midwest** | **South** | **West** |
| **1999** | 3.58 (3.32–3.84) | 4.34 (4.07–4.61) | 4.07 (3.85–4.28) | 3.18 (2.92–3.44) |
| **2000** | 6.15 (5.82–6.49) | 7.50 (7.15–7.85) | 5.89 (5.63–6.15) | 7.12 (6.74–7.51) |
| **2001** | 6.61 (6.27–6.96) | 8.05 (7.68–8.41) | 5.83 (5.57–6.09) | 7.31 (6.93–7.69) |
| **2002** | 6.62 (6.28–6.97) | 8.62 (8.24–8.99) | 6.22 (5.95–6.48) | 7.46 (7.08–7.85) |
| **2003** | 6.82 (6.47–7.17) | 8.82 (8.44–9.20) | 7.07 (6.79–7.36) | 8.08 (7.68–8.47) |
| **2004** | 7.05 (6.69–7.40) | 9.29 (8.91–9.68) | 6.99 (6.72–7.27) | 8.41 (8.01–8.80) |
| **2005** | 7.11 (6.76–7.46) | 9.76 (9.37–10.15) | 7.25 (6.97–7.53) | 8.15 (7.76–8.53) |
| **2006** | 6.77 (6.43–7.12) | 8.74 (8.38–9.11) | 6.86 (6.60–7.13) | 7.58 (7.21–7.95) |
| **2007** | 7.43 (7.07–7.79) | 8.79 (8.43–9.16) | 7.19 (6.91–7.46) | 7.37 (7.01–7.73) |
| **2008** | 7.27 (6.91–7.62) | 9.08 (8.71–9.46) | 7.21 (6.94–7.48) | 7.82 (7.45–8.18) |
| **2009** | 7.08 (6.73–7.42) | 8.70 (8.34–9.06) | 7.34 (7.07–7.61) | 7.28 (6.93–7.63) |
| **2010** | 7.83 (7.47–8.19) | 8.94 (8.58–9.31) | 8.01 (7.73–8.29) | 7.94 (7.58–8.30) |
| **2011** | 8.21 (7.84–8.58) | 9.51 (9.14–9.89) | 7.97 (7.69–8.24) | 7.86 (7.51–8.22) |
| **2012** | 7.66 (7.31–8.02) | 9.53 (9.16–9.90) | 8.16 (7.89–8.44) | 8.32 (7.96–8.68) |
| **2013** | 8.29 (7.92–8.66) | 9.65 (9.28–10.03) | 8.10 (7.83–8.37) | 7.61 (7.27–7.95) |
| **2014** | 8.15 (7.78–8.51) | 10.02 (9.64–10.40) | 7.60 (7.34–7.86) | 7.11 (6.79–7.44) |
| **2015** | 8.08 (7.72–8.44) | 9.44 (9.08–9.81) | 7.50 (7.25–7.75) | 7.14 (6.82–7.46) |
| **2016** | 7.91 (7.55–8.26) | 9.47 (9.10–9.83) | 7.50 (7.25–7.75) | 7.17 (6.85–7.48) |
| **2017** | 8.23 (7.88–8.59) | 9.39 (9.03–9.74) | 8.11 (7.85–8.36) | 7.29 (6.98–7.61) |
| **2018** | 8.41 (8.05–8.77) | 9.93 (9.57–10.29) | 8.35 (8.10–8.61) | 7.10 (6.80–7.41) |
| **2019** | 8.25 (7.90–8.60) | 9.76 (9.40–10.11) | 8.29 (8.04–8.54) | 7.37 (7.06–7.67) |
| **2020** | 10.90 (10.50–11.30) | 12.44 (12.04–12.84) | 10.85 (10.57–11.13) | 8.69 (8.36–9.02) |
| **2021** | 9.11 (8.74–9.48) | 10.58 (10.20–10.95) | 10.60 (10.31–10.88) | 8.78 (8.44–9.11) |
| **2022** | 8.32 (7.98–8.66) | 9.90 (9.54–10.25) | 10.16 (9.89–10.43) | 7.77 (7.46–8.07) |
| **2023** | 7.62 (7.29–7.95) | 9.37 (9.03–9.72) | 9.39 (9.13–9.64) | 6.79 (6.51–7.08) |

**Supplemental Table 6:** State- trends in Parkinson’s disease and associated mental health disorders related mortality, shown as age-adjusted mortality rates per 100,000 among adults aged ≥45 in the United States, 1999–2023.

| **States** | **Age Adjusted Mortality Rate (95% CI)** | |
| --- | --- | --- |
|  | **1999-2020** | **2021-2023** |
| **Alabama** | 6.19 (5.94–6.44) | 5.92 (5.33–6.51) |
| **Alaska** | 9.23 (8.07–10.39) | 8.68 (6.38–11.55) |
| **Arizona** | 5.30 (5.10–5.50) | 5.59 (5.14–6.05) |
| **Arkansas** | 5.99 (5.68–6.29) | 9.74 (8.77–10.71) |
| **California** | 6.84 (6.74–6.93) | 6.20 (5.97–6.42) |
| **Colorado** | 9.49 (9.15–9.82) | 13.64 (12.72–14.55) |
| **Connecticut** | 8.19 (7.88–8.49) | 8.20 (7.41–8.99) |
| **Delaware** | 7.53 (6.91–8.15) | 10.09 (8.45–11.72) |
| **District of Columbia** | 4.93 (4.29–5.58) | 7.48 (5.53–9.89) |
| **Florida** | 5.83 (5.72–5.93) | 8.72 (8.42–9.02) |
| **Georgia** | 6.53 (6.32–6.74) | 7.62 (7.11–8.12) |
| **Hawaii** | 6.66 (6.22–7.11) | 5.53 (4.59–6.48) |
| **Idaho** | 8.74 (8.20–9.28) | 10.65 (9.29–12.00) |
| **Illinois** | 7.69 (7.52–7.86) | 7.21 (6.79–7.62) |
| **Indiana** | 9.15 (8.89–9.41) | 10.65 (9.95–11.35) |
| **Iowa** | 9.70 (9.35–10.06) | 11.43 (10.43–12.43) |
| **Kansas** | 8.61 (8.24–8.97) | 10.45 (9.41–11.49) |
| **Kentucky** | 9.41 (9.08–9.73) | 18.18 (17.06–19.31) |
| **Louisiana** | 4.76 (4.53–5.00) | 6.81 (6.12–7.51) |
| **Maine** | 10.21 (9.67–10.76) | 9.25 (7.99–10.52) |
| **Maryland** | 9.98 (9.68–10.27) | 12.16 (11.38–12.94) |
| **Massachusetts** | 6.84 (6.63–7.05) | 8.91 (8.31–9.51) |
| **Michigan** | 8.23 (8.04–8.43) | 8.26 (7.77–8.75) |
| **Minnesota** | 12.43 (12.11–12.76) | 15.72 (14.81–16.62) |
| **Mississippi** | 6.61 (6.28–6.95) | 10.54 (9.49–11.60) |
| **Missouri** | 7.52 (7.28–7.75) | 8.94 (8.29–9.59) |
| **Montana** | 7.29 (6.73–7.85) | 8.45 (7.00–9.90) |
| **Nebraska** | 11.53 (11.01–12.06) | 15.54 (13.98–17.10) |
| **Nevada** | 4.84 (4.51–5.17) | 6.38 (5.56–7.20) |
| **New Hampshire** | 9.90 (9.32–10.49) | 11.62 (10.11–13.13) |
| **New Jersey** | 6.63 (6.44–6.81) | 6.71 (6.25–7.16) |
| **New Mexico** | 7.89 (7.45–8.33) | 6.45 (5.52–7.37) |
| **New York** | 6.79 (6.66–6.91) | 7.67 (7.35–8.00) |
| **North Carolina** | 8.57 (8.35–8.78) | 9.93 (9.39–10.48) |
| **North Dakota** | 10.47 (9.69–11.25) | 11.20 (9.10–13.30) |
| **Ohio** | 9.99 (9.80–10.19) | 9.32 (8.85–9.80) |
| **Oklahoma** | 8.38 (8.05–8.70) | 12.78 (11.77–13.79) |
| **Oregon** | 10.78 (10.43–11.13) | 14.83 (13.83–15.84) |
| **Pennsylvania** | 8.50 (8.34–8.66) | 9.07 (8.64–9.49) |
| **Rhode Island** | 9.20 (8.62–9.79) | 13.69 (11.83–15.54) |
| **South Carolina** | 9.10 (8.78–9.41) | 14.32 (13.43–15.22) |
| **South Dakota** | 8.62 (7.96–9.27) | 9.71 (7.91–11.50) |
| **Tennessee** | 9.01 (8.75–9.28) | 11.27 (10.56–11.98) |
| **Texas** | 8.91 (8.76–9.06) | 10.46 (10.08–10.84) |
| **Utah** | 7.78 (7.33–8.23) | 6.88 (5.90–7.85) |
| **Vermont** | 11.86 (10.96–12.75) | 10.73 (8.67–12.79) |
| **Virginia** | 7.41 (7.19–7.63) | 9.68 (9.08–10.27) |
| **Washington** | 10.01 (9.74–10.29) | 10.37 (9.70–11.03) |
| **West Virginia** | 8.73 (8.29–9.17) | 10.13 (8.92–11.34) |
| **Wisconsin** | 9.85 (9.58–10.12) | 11.51 (10.75–12.26) |
| **Wyoming** | 6.15 (5.40–6.91) | 9.23 (7.14–11.74) |

**Supplemental Table 7:** State- trends in Parkinson’s disease and associated mental health disorders related mortality, shown as absolute number of deaths among adults aged ≥45 in the United States, 1999–2023.

| **States** | **Deaths** | | | |
| --- | --- | --- | --- | --- |
|  | **1999-2020** | **2021-2023** | **1999-2023** | **%** |
| **Alabama** | 2399 | 394 | 2793 | 1.17% |
| **Alaska** | 254 | 49 | 303 | 0.13% |
| **Arizona** | 2800 | 583 | 3383 | 1.42% |
| **Arkansas** | 1504 | 392 | 1896 | 0.80% |
| **California** | 18510 | 2885 | 21395 | 8.98% |
| **Colorado** | 3126 | 862 | 3988 | 1.67% |
| **Connecticut** | 2811 | 423 | 3234 | 1.36% |
| **Delaware** | 577 | 149 | 726 | 0.30% |
| **District of Columbia** | 225 | 50 | 275 | 0.12% |
| **Florida** | 12205 | 3323 | 15528 | 6.51% |
| **Georgia** | 3875 | 884 | 4759 | 2.00% |
| **Hawaii** | 871 | 135 | 1006 | 0.42% |
| **Idaho** | 1018 | 242 | 1260 | 0.53% |
| **Illinois** | 7978 | 1169 | 9147 | 3.84% |
| **Indiana** | 4794 | 893 | 5687 | 2.39% |
| **Iowa** | 3001 | 508 | 3509 | 1.47% |
| **Kansas** | 2170 | 394 | 2564 | 1.08% |
| **Kentucky** | 3199 | 1020 | 4219 | 1.77% |
| **Louisiana** | 1608 | 381 | 1989 | 0.83% |
| **Maine** | 1341 | 206 | 1547 | 0.65% |
| **Maryland** | 4374 | 944 | 5318 | 2.23% |
| **Massachusetts** | 4167 | 846 | 5013 | 2.10% |
| **Michigan** | 7012 | 1116 | 8128 | 3.41% |
| **Minnesota** | 5619 | 1175 | 6794 | 2.85% |
| **Mississippi** | 1501 | 390 | 1891 | 0.79% |
| **Missouri** | 3946 | 737 | 4683 | 1.96% |
| **Montana** | 658 | 132 | 790 | 0.33% |
| **Nebraska** | 1901 | 386 | 2287 | 0.96% |
| **Nevada** | 838 | 238 | 1076 | 0.45% |
| **New Hampshire** | 1106 | 229 | 1335 | 0.56% |
| **New Jersey** | 5120 | 833 | 5953 | 2.50% |
| **New Mexico** | 1247 | 189 | 1436 | 0.60% |
| **New York** | 11623 | 2132 | 13755 | 5.77% |
| **North Carolina** | 6214 | 1312 | 7526 | 3.16% |
| **North Dakota** | 714 | 113 | 827 | 0.35% |
| **Ohio** | 10216 | 1483 | 11699 | 4.91% |
| **Oklahoma** | 2557 | 624 | 3181 | 1.33% |
| **Oregon** | 3624 | 853 | 4477 | 1.88% |
| **Pennsylvania** | 11126 | 1756 | 12882 | 5.41% |
| **Rhode Island** | 987 | 212 | 1199 | 0.50% |
| **South Carolina** | 3269 | 1007 | 4276 | 1.79% |
| **South Dakota** | 681 | 115 | 796 | 0.33% |
| **Tennessee** | 4487 | 980 | 5467 | 2.29% |
| **Texas** | 13774 | 2977 | 16751 | 7.03% |
| **Utah** | 1157 | 196 | 1353 | 0.57% |
| **Vermont** | 675 | 105 | 780 | 0.33% |
| **Virginia** | 4321 | 1029 | 5350 | 2.24% |
| **Washington** | 5127 | 948 | 6075 | 2.55% |
| **West Virginia** | 1546 | 273 | 1819 | 0.76% |
| **Wisconsin** | 5024 | 904 | 5928 | 2.49% |
| **Wyoming** | 258 | 67 | 325 | 0.14% |
| **Total** | 199135 | 39243 | 238378 | 100 % |

**Supplemental Table 8:** Percentage distribution of place of death in the United States (1999–2023), with Parkinson’s disease and associated mental health disorders related mortality highlighted.

| **Place of death** | **Deaths** | **% of Death** |
| --- | --- | --- |
| **Medical Facility** | 36905 | 15.48% |
| **Decedent's home** | 50177 | 21.04% |
| **Hospice facility** | 11772 | 4.94% |
| **Nursing home/long term care** | 125511 | 52.64% |
| **Other/ Place of death unknown** | 14011 | 5.88% |
| **Total** | 238376 | 100.00% |

**Supplementary Figure 1:** State stratified age-adjusted mortality rates per 100,000 for Parkinson’s disease and associated mental health disorders related mortality in United States adults aged ≥45 years, 1999-2023.


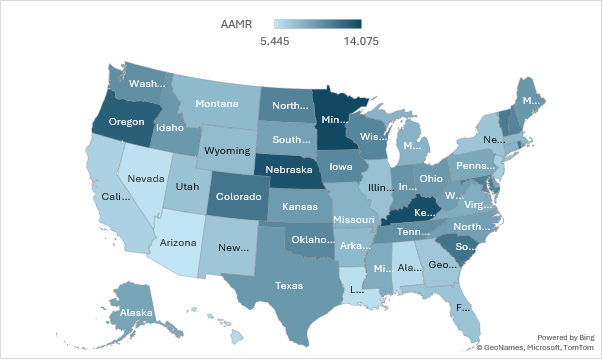


**Supplementary Figure 2:** Percentage distribution of place of death in the United States (1999–2023), with Parkinson’s disease and associated mental health disorders related mortality highlighted.

**
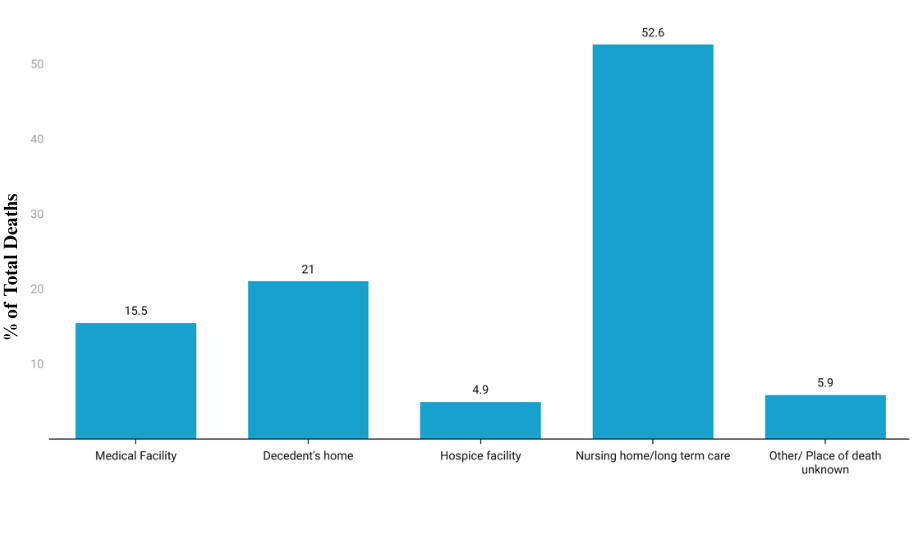
**
